# Research on Pork Cut and Freshness Determination Method Based on Computer Vision

**DOI:** 10.3390/foods13243986

**Published:** 2024-12-10

**Authors:** Shihao Song, Qiqi Guo, Xiaosa Duan, Xiaojing Shi, Zhenyu Liu

**Affiliations:** 1 School of Information Science and Engineering, Shanxi Agricultural University, Jinzhong 030801, China; ssh0901@163.com; 2 College of Agricultural Engineering, Shanxi Agricultural University, Jinzhong 030801, China; exam8800@126.com (Q.G.); 18235947970@163.com (X.D.); 3 College of Food Science and Engineering, Shanxi Agricultural University, Jinzhong 030801, China; sxj_hn@163.com; 4 Dryland Farm Machinery Key Technology and Equipment Key Laboratory of Shanxi Province, Jinzhong 030801, China

**Keywords:** convolutional neural networks, computer vision, pork cuts, meat quality detection, food safety

## Abstract

With the increasing importance of meat quality inspection, traditional manual evaluation methods face challenges in terms of efficiency and accuracy. To improve the precision and efficiency of pork quality assessment, an automated detection method based on computer vision technology is proposed for evaluating different parts and freshness of pork. First, high-resolution cameras were used to capture image data of Jinfen white pigs, covering three pork cuts—hind leg, loin, and belly—across three different collection times. These three parts were categorized into nine datasets, and the sample set was expanded through digital image processing techniques. Next, five convolutional neural network models—VGGNet, ResNet, DenseNet, MobileNet, and EfficientNet—were selected for feature recognition experiments. The experimental results showed that the MobileNetV3_Small model achieved an accuracy of 98.59%, outperforming other classical network architectures while being more lightweight. Further statistical analysis revealed that the *p*-values for ResNet101, EfficientNetB0, and EfficientNetB1 were all greater than 0.05, indicating that the performance differences between these models and MobileNetV3_Small were not statistically significant. In contrast, other models showed significant performance differences (*p*-value < 0.05). Finally, based on the PYQT5 framework, the MobileNetV3_Small model was deployed on a local client, realizing an efficient and accurate end-to-end automatic recognition system. These findings can be used to effectively enhance the efficiency and reliability of pork quality detection, providing a solid foundation for the development of pork safety monitoring systems.

## 1. Introduction

With rapid economic development and the continuous improvement in people’s living standards, consumer demands for pork quality have been steadily increasing, with greater attention being paid to the freshness of meat products. This is closely related to public health, especially concerning pork, which is the most consumed meat in the market [1]. Traditionally, pork cut identification and freshness assessment have primarily relied on manual inspection, which is subjective, labor-intensive, and time-consuming. With continuous advancements in image processing technology and image analysis methods, the application of computer vision technology in agricultural products and food detection has been growing. To enhance the efficiency and accuracy of pork quality control, researchers have started exploring computer vision-based methods for pork cut identification and freshness determination [2].

In recent years, research institutions and researchers, both domestically and internationally, have introduced modern detection methods, such as electronic noses, computer vision, and near-infrared spectroscopy, into pork detection. However, near-infrared spectroscopy analyzers and electronic nose devices are expensive, and the electronic nose detection method is relatively complex, making it difficult to implement in practical detection tasks. In contrast, computer vision detection methods are relatively low in cost and easy to promote and apply. In the evaluation of meat quality characteristics, computer vision is considered the most promising objective evaluation method. This approach can automate, quickly, and objectively perform pork cut identification and freshness assessment, thereby improving production efficiency, reducing costs, and ensuring food quality and safety [3]. These methods utilize image processing, machine learning, and deep learning techniques to analyze features in pork images, such as color, texture, and shape, to identify pork cuts and determine freshness [4,5,6,7]. Most importantly, through visualization and digitization, the cut and freshness information of pork can be tracked and recorded, which aids in traceability and quality management [8]. Moreover, this research also provides new quality control measures for meat production enterprises. These technologies can be integrated with other advanced quality monitoring systems to achieve comprehensive quality management and rapid response. At the same time, further improvements and optimizations of algorithms and models can adapt to different production environments and changes, enhancing the accuracy and stability of the algorithms [9].

In order to improve the real-time performance of pork freshness detection, Qiu Hongtao et al. proposed a new method for pork freshness grading based on the Caffe framework and residual neural networks. This method ensures high grading accuracy while maintaining a simple, real-time, and non-destructive detection process, making it a more efficient approach for pork freshness grading [10]. Qu Shihai et al. developed a novel pork freshness grading system utilizing photoelectric detection technology, image processing analysis, and pattern recognition techniques that allows for more accurate identification of pork freshness. The image processing component extracts color information from the H, S, and I channels of the HSI color model and uses a BP neural network to classify the test set, achieving the goal of pork freshness grading [11]. In order to find a fast and accurate method for objectively assessing pork meat color, Sun Jingxin et al. conducted a study on digital image processing techniques and stepwise regression models. The research indicated that digital image processing technology can effectively and objectively evaluate the color of chilled pork [12]. Zhang et al. conducted a comparative analysis of six CNN models—AlexNet, GoogLeNet, ResNet18, DarkNet19, SqueezeNet, and VGG16—on their effectiveness in recognizing the shape features of different meat slices and adulterated mutton. The results demonstrated that the ResNet-18 model has superior learning capability for distinguishing authentic and counterfeit mutton images [13]. Building on the previous work, Xu Yuting et al. incorporated machine learning techniques, utilizing an SVM classification model for pork cut recognition. The SVM algorithm addressed the issue of high-dimensional spectral data, effectively improving classification accuracy [14].

The main content of this study was divided into the following parts: (1) reviewing and analyzing the current research status in related fields both domestically and internationally; (2) collecting images using cameras and establishing an image dataset suitable for the determination of pork parts and freshness, followed by image analysis; (3) comparing the performance of various convolutional neural network models for this task, ultimately selecting the MobileNetV3_Small model, which demonstrated the best performance; (4) constructing and optimizing the MobileNetV3_Small model for pork part and freshness determination and performing feature visualization to analyze the effectiveness of the improved model in extracting pork characteristics; and (5) designing and implementing a user-friendly interactive interface based on the MobileNetV3_Small model [15].

## 2. Materials and Methods

### 2.1. Sample Preparation

In this study, the Jinfen white pig, a locally bred pig from Shanxi, was selected as the subject for imaging. The Jinfen white pig is a lean-type breed developed by Shanxi Agricultural University over more than 20 years. It incorporates superior genetic traits from four renowned pig breeds, both domestic and international—Large White, Landrace, Berkshire, and Taihu—exhibiting exceptional overall performance. The pork cut classification method employed was the standard approach used by the majority of pork processing enterprises in China, where cuts are made based on different parts of the pig carcass. The dataset collected can thus reflect the image characteristics of most pork cuts to a certain extent.

The data collection process was conducted in a dedicated image acquisition laboratory, where the lighting conditions were kept constant to minimize the interference of external light fluctuations. To ensure image quality, a high-resolution camera, the Canon EOS 5D mark IV, was used, and a tripod was employed to maintain the stability of the camera during each shot. During the capture process, the camera was positioned at a fixed distance and angle relative to the pork sample to ensure consistency across all images. All samples were photographed against the same neutral white background to prevent color deviations from affecting the image analysis. To ensure the diversity and comprehensiveness of the data, different lighting modes (a combination of natural light and artificial light sources) were used, while avoiding excessive shadows or reflections.

**Figure 1:**
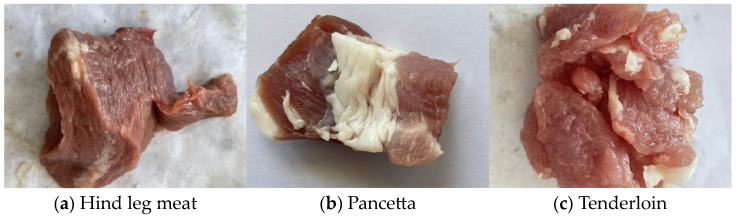
Images of different parts of pork collected.

A total of 2757 pork images were captured, covering three distinct cuts: ham (H), loin (L), and belly (M). To ensure a diverse set of sample data, the images were captured gradually at intervals of 6 to 12 h. The captured images were then categorized into 9 groups based on the time of acquisition, and the dataset was randomly split into training, validation, and test sets with a ratio of 8:1:1. Detailed information about the initial dataset is provided in Table 1 and Figure 1. In Figure 1, (a) represents the ham images taken from 0 to 6 h, (b) shows the belly images from 0 to 6 h, and (c) depicts the loin images from 0 to 6 h [8].

### 2.2. Expansion of the Dataset

The robustness of convolutional neural network (CNN) models relies on a large number of training samples. Insufficient data can lead to overfitting during model training, resulting in poor generalization ability and limiting the model’s practical applicability. An increase in sample data allows the model to learn more information, thereby improving its resistance to interference. Given that manual sample collection is time-consuming and labor-intensive, this study employed data augmentation techniques, such as digital image processing algorithms, to expand the dataset.

The brightness, sharpness, and contrast of images can be adjusted to simulate the effects of lighting factors on photography. In the real world, lighting conditions can change, such as variations between daytime and nighttime or sunny and cloudy weather. These changes affect the brightness and contrast of images. By adjusting the brightness, sharpness, and contrast, augmentation algorithms can simulate these variations, helping the model better adapt to images captured under different lighting conditions. This data augmentation improves a model’s robustness in low-light or high-light environments, preventing interference from unstable lighting in practical applications.

Adding Gaussian noise can simulate the effect of images taken in foggy weather. Noise, especially Gaussian noise, mimics the impact of harsh environmental conditions such as haze, rainy weather, or low visibility. These factors lead to blurred images, loss of details, or color changes. By adding noise, the augmented data enable a model to learn how to handle images captured under these complex conditions, thereby enhancing the model’s stability and accuracy.

In actual photography, the relative position between the camera and the subject affects the angle, perspective, and orientation of the image. By rotating the image (e.g., 180° clockwise) and applying horizontal or vertical symmetry transformations, different angles and orientations of the image can be simulated. This allows the model to see images from various viewpoints during training, improving its adaptability to different shooting angles and reducing over-reliance on specific perspectives.

**Figure 2:**
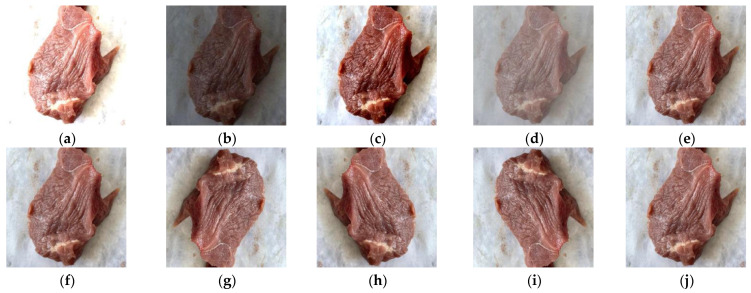
Example of pork pictures after data expansion. (**a**) Elevated brightness. (**b**) Loss of brightness. (**c**) Elevated contrast. (**d**) Reduced contrast. (**e**) Increased sharpening. (**f**) Reduced sharpening. (**g**) Rotated 180°. (**h**) Horizontal. (**i**) Vertical mirror symmetry. (**j**) With Gaussian noise added.

Using digital image processing techniques to simulate various influencing factors during data acquisition for different parts of pork, a single image can generate 10 derived images. Figure 2 demonstrates examples of data augmentation. Parts (a) and (b) represent brightness enhancement and reduction, respectively; (c) and (d) represent contrast enhancement and reduction, respectively; (e) and (f) represent sharpening enhancement and reduction, respectively; (g) shows a 180° rotation of the original image; (h) and (i) correspond to horizontal and vertical flip transformations, respectively; and (j) represents the addition of Gaussian noise.

Detailed information following data augmentation is shown in Table 2, where the total number of images has been increased from 2757 to 30,327.

### 2.3. Classical Convolutional Neural Network Model Architecture

#### 2.3.1. VGGNet

**Figure 3:**
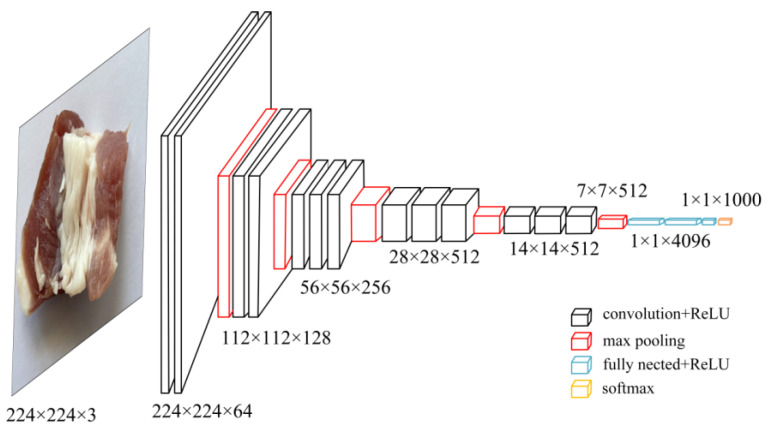
Structure of VGG16 model.

The VGG model has a large number of layers, but a simpler structure. Figure 3 illustrates the architecture of the VGG16 network model. The VGG model uses 3 × 3 convolutional kernels, replacing larger- kernels with consecutive 3 × 3 convolutions while keeping the convolution stride fixed at 1. This increases the depth of the network, adds more nonlinear layers, enables the learning of more complex features, and significantly reduces the number of model parameters [16].

#### 2.3.2. ResNet

**Figure 4:**
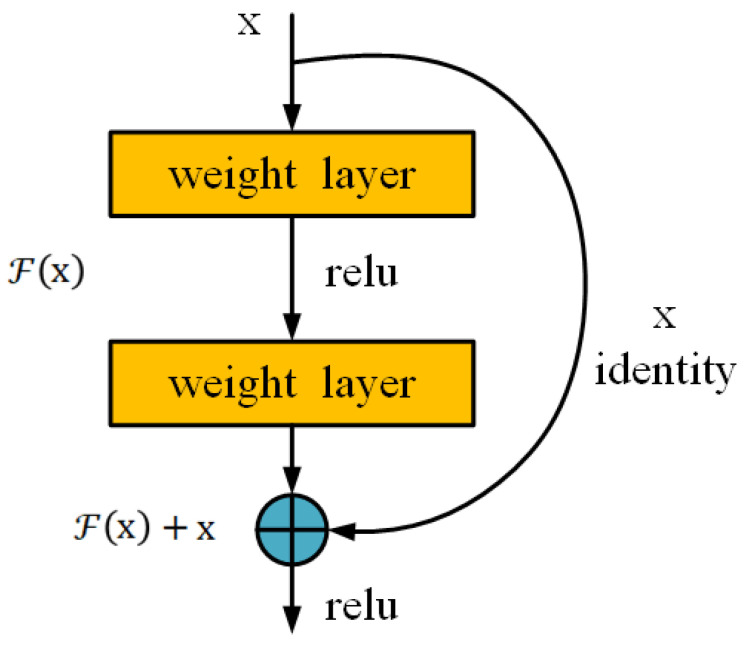
Residual learning unit.

ResNet (Residual Network) is a deep convolutional neural network model that effectively improves the performance and training speed of deep CNNs by introducing “residual blocks” [17]. The model is primarily composed of multiple residual blocks, each containing several convolutional layers, batch normalization layers, and a shortcut connection. The network is structured into several stages, with each stage consisting of multiple residual blocks. As the network depth increases, the spatial dimensions of the feature maps gradually decrease, while the number of channels increases to extract more complex features. Additionally, the network ends with a global average pooling layer followed by a softmax classifier. The shortcut connection within the residual block directly adds the input signal to the output, as shown in Figure 4. This shortcut connection can be viewed as a “skip connection”, which improves gradient flow, mitigates the vanishing or exploding gradient problem, and facilitates network optimization.

#### 2.3.3. DenseNet

**Figure 5:**
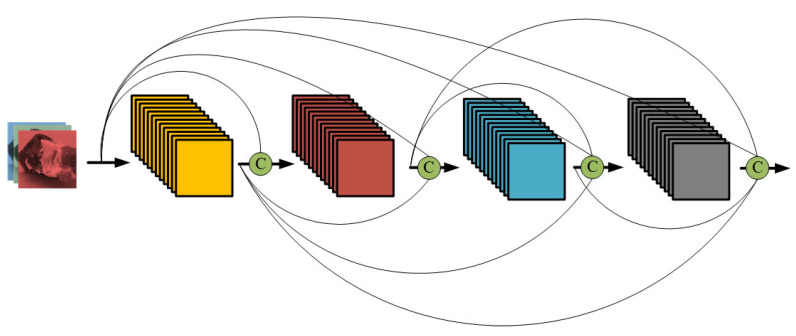
Dense connectivity mechanism of DenseNet.

DenseNet (densely connected convolutional network) is similar to ResNet in that it addresses the vanishing and exploding gradient problems in deep network training by introducing interlayer connections. However, unlike ResNet, DenseNet employs dense connections [18]. The model is primarily composed of alternating dense blocks and transition layers, with each dense block consisting of multiple convolutional layers and batch normalization layers, as shown in Figure 5. In DenseNet, each layer is connected to all previous layers, enhancing feature propagation and reuse while preventing information loss. Additionally, it incorporates transition layers, which use convolution and pooling layers to control the reduction in feature map size and the number of channels. The core idea of DenseNet is dense connectivity, which improves the model’s performance and training speed. It also utilizes batch normalization and ReLU activation functions to accelerate model convergence and enhance robustness.

#### 2.3.4. MobileNet

**Figure 6:**
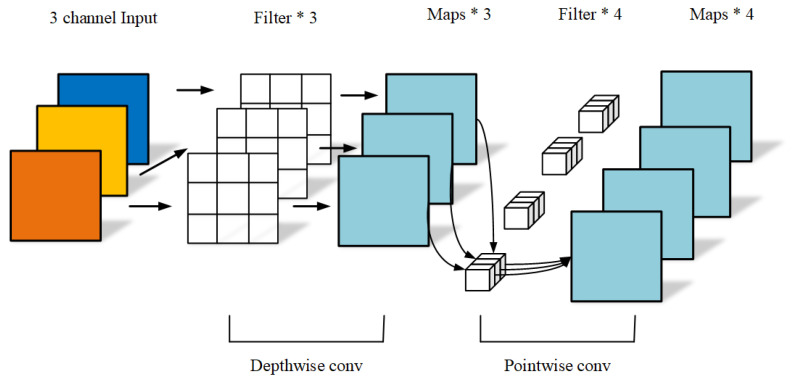
Schematic representation of the depthwise separable convolution.

MobileNet is a lightweight convolutional neural network developed by Google that is designed to provide efficient computation speed and smaller models when running on mobile devices [19]. MobileNet is primarily composed of depthwise separable convolutions and 1 × 1 convolutions. Depthwise separable convolution is divided into two steps: first, performing a convolution on each input channel, then applying a 1 × 1 convolution to mix the channels, followed by pointwise convolutions. Figure 6 illustrates the concept of depthwise separable convolution. MobileNet is a lightweight network that reduces the number of parameters and computational cost by using depthwise separable convolutions, and aggregates channel information through global average pooling.

#### 2.3.5. EfficientNet

**Figure 7:**
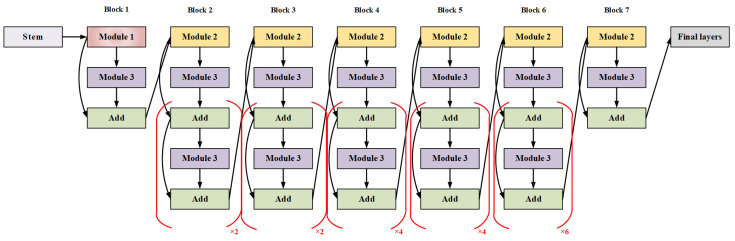
Schematic diagram of EfficientNet structure.

EfficientNet is a convolutional neural network model developed by Google that improves both model performance and efficiency through innovative design. The model adopts a residual network architecture and incorporates the MBConv (mobile inverted residual bottleneck) module and the SE (squeeze and excitation) module. It utilizes an adaptive network scaling method to automatically determine the network’s depth, width, and resolution, thereby enhancing performance without increasing computational cost. EfficientNet consists of 7 stages, with each stage containing a set of MBConv modules and an SE module. Each MBConv module includes depthwise convolution, expansion convolution, and projection convolution, which are used to extract features at different levels. The SE module is employed to reweight feature channels, thereby enhancing the model’s expressive power [20]. Figure 7 illustrates the structure of EfficientNet. A complexity coefficient, denoted phi, is used to generate models of varying complexity by adjusting the value of phi, resulting in models ranging from EfficientNet-B0 to EfficientNet-B7. EfficientNet has achieved outstanding performance across multiple image classification and object detection tasks.

### 2.4. Dropout Strategy

**Figure 8:**
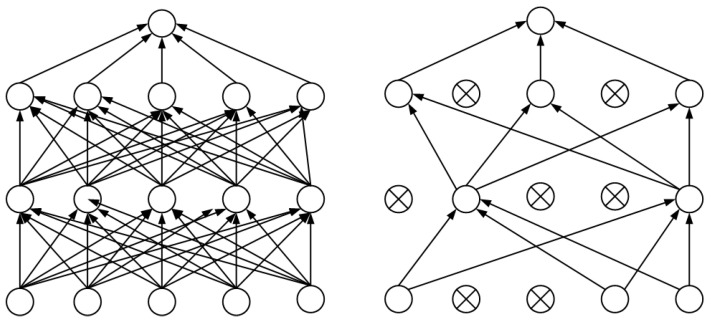
Dropout schematic.

In this study, the dropout strategy is introduced into the classical convolutional neural network architecture to improve the model’s recognition performance and generalization ability. Dropout works by randomly setting the output of neurons to zero with a certain probability, preventing neurons from participating in forward or backward propagation, thereby increasing the model’s robustness. A schematic of the dropout strategy is shown in Figure 8. With each training iteration, the model structure changes, reducing the dependency on individual neurons and effectively preventing overfitting and enhancing generalization. By randomly deactivating neurons, dropout makes the network more robust to variations in input data and reduces model complexity [21].

### 2.5. Self-Attention Mechanisms

Introducing the self-attention mechanism into convolutional neural networks (CNNs) can enhance a model’s performance and feature representation capabilities. Traditional CNNs typically apply the same weights to all input positions, which may limit their ability to handle complex scenarios and long-range dependencies. The self-attention mechanism enables each input element to interact with others and assign weights, thereby better capturing long-range dependencies and improving model performance on complex tasks. The multiplicative attention mechanism, which is the core of the self-attention module, effectively reflects the relationships between input elements [22].

In multiplicative attention, this is achieved through the operation of three matrices: query (Q), key (K), and value (V). First, each element of the input sequence is transformed into a vector representation through an embedding layer, forming a matrix (X). The input matrix (X) is then mapped to the query matrix (Q), key matrix (K), and value matrix (V) using three linear transformations:

(1) $$Q=XW_{Q},K=XW_{K},V=XW_{V}$$

where (W_Q_), (W_K_), and (W_V_) are the learned weight matrices.

The dot product between the query and key is then computed:

(2) $$scores=QK^{T}$$

To prevent the values from becoming excessively large, the dot product is scaled by the dimension of the key, denoted d_k_:

(3) $$scaled\_scores=\frac{scores}{\sqrt{d_{k}}}$$

The scores are then converted into attention weights using the softmax function:

(4) attention_weights=softmax(scaled_scores)

The attention weights are then applied to the value matrix (V), resulting in the final output:

(5) output=attention_weightsV

In summary, the complete multiplicative attention mechanism can be represented by the following formula:

(6) $$output=soft\max(\frac{QK^{T}}{\sqrt{d_{k}}})V$$

This mechanism enables the model to capture long-range dependencies, as it considers information from all positions simultaneously without being constrained by a sequential structure.

### 2.6. Hardware and Software Environment Configuration

The training and testing of all models in this study were conducted on a Microsoft Windows 11 operating system. The CPU was an AMD Ryzen 7 5800H with Radeon graphics, and the GPU wass a GeForce RTX 3070, with 8192 MiB of available GPU memory. The deep learning frameworks used in the experiments were TensorFlow and Keras. The specific details of the software and hardware configurations of the experimental machine are provided in Table 3.

### 2.7. Model Training Strategy

In the experiments, hyperparameters during model training were standardized to compare the performance of different models. The gradient optimization algorithm used during training was Adam, with a learning rate set to 0.001 and 100 epochs. Cross-entropy was chosen as the loss function.

### 2.8. Evaluation Indicators

The experimental dataset was divided into three parts: the training set, validation set, and test set. Only the data from the training set were used for model training. After each training epoch, the model’s performance metrics were evaluated using the validation set. By analyzing the training data from each epoch, the model’s convergence rate and other related information could be observed. Finally, the test set was used for the final evaluation of the model’s performance.

The model’s performance on the test set can be evaluated using metrics such as accuracy, precision, recall, and F1 score [23]. Here, TP (true positive) represents the number of positive samples correctly classified as positive, TN (true negative) represents the number of negative samples correctly classified as negative, FP (false positive) represents the number of negative samples incorrectly classified as positive, and FN (false negative) represents the number of positive samples incorrectly classified as negative. Metrics such as accuracy can be calculated using the following formulas:

(7) $$accuracy=\frac{TP+TN}{TP+TN+FP+FN}$$

(8) $$precision=\frac{TP}{TP+FP}$$

(9) $$recall=\frac{TP}{TP+FN}$$

(10) $$F1\;score=\frac{2\times precision\times recall}{precision+recall}=\frac{2\times TP}{2\times TP+FN+FP}$$

Calculation of the above metrics was performed for each class, and the overall values for the model were taken as the average of the metrics across all classes.

### 2.9. Significance Analysis

In significance analysis, it is crucial to select an appropriate statistical method to assess the differences between models. Hypothesis testing is a commonly used approach. When performing hypothesis testing, an initial null hypothesis and an alternative hypothesis are established. The null hypothesis indicates that there is no significant difference between two samples or groups, while the alternative hypothesis suggests that there is a significant difference. To compare the predictive results of two models for significant differences, the *p*-value is typically used as the validation criterion. To ensure the validity of the statistical tests, it is essential to apply suitable statistical methods, such as the *t*-test, analysis of variance (ANOVA), or non-parametric tests. In this study, a paired t-test was employed to compare the accuracy of different models. If the *p*-value ≤ α (usually α = 0.05), the null hypothesis is rejected and the alternative hypothesis is accepted, indicating a significant difference between the two groups of models. If the *p*-value > α, the null hypothesis cannot be rejected, suggesting insufficient evidence to conclude a significant difference between the two groups [24].

## 3. Results and Discussion

### 3.1. Model Comparison

**Figure 9:**
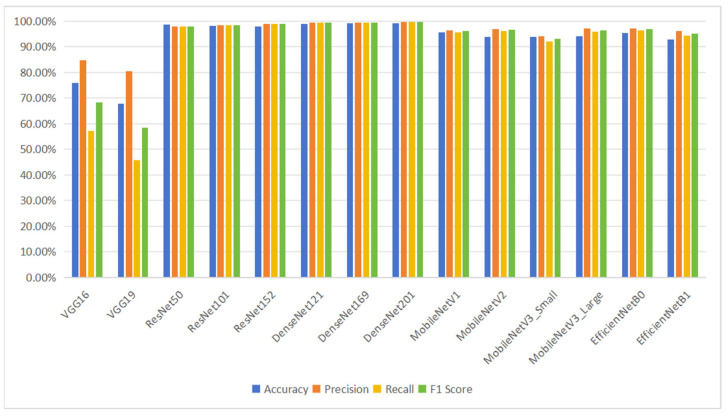
CNN model results.

This section presents a comparative experimental analysis of the convolutional neural network models VGG, ResNet, DenseNet, MobileNet, and EfficientNet. Specifically, VGG comprises two architectures, VGG16 and VGG19; ResNet comprises three architectures, ResNet50, ResNet101, and ResNet152; DenseNet comprises three architectures, DenseNet121, DenseNet169, and DenseNet201; MobileNet comprises four architectures, MobileNetV1, MobileNetV2, MobileNetV3_Small, and MobileNetV3_Large; and EfficientNet comprises two architectures, EfficientNetB0 and EfficientNetB7. Table 4 shows the final test results of each model on the test set, and Figure 9 presents a bar chart comparing the accuracy, precision, recall, and F1 score of the various models.

The VGG model uses 3 × 3 convolutional kernels and replaces larger kernels with consecutive 3 × 3 kernels while fixing the convolutional stride to 1. This design aims to deepen the network, increase the number of nonlinear layers, and learn more complex features, all while maintaining a consistent receptive field and significantly reducing the number of model parameters. The final results showed that the VGG16 model achieved an accuracy of 75.81% on the test set, while the VGG19 model achieved an accuracy of 67.87% on the test set.

The ResNet series models Introduce the concepts of skip connections and residual learning, which facilitate model optimization. The residual blocks within the model use skip connections to alleviate the vanishing gradient problem that arises when increasing the depth of deep neural networks. The final results showed that ResNet50 achieved an accuracy of 98.56%, ResNet101 achieved an accuracy of 98.19%, and ResNet152 achieved an accuracy of 97.83%.

The DenseNet model differs from other models that improve CNN performance by either increasing network depth (e.g., ResNet) or widening the network structure (e.g., Inception). Instead, DenseNet enhances performance and reduces model parameters through feature reuse and bypass connections. Unlike the skip connections in ResNet, DenseNet employs a dense connectivity strategy, where all feature maps from preceding layers are used as inputs to the current layer, and the feature maps from the current layer are passed as inputs to all subsequent layers. This strategy maximizes the flow of information between the layers. The final results showed that DenseNet121 achieved a final accuracy of 98.92%, DenseNet169 achieved a final accuracy of 99.28%, and DenseNet201 achieved a final accuracy of 99.28%.

The MobileNet series models are compact and efficient CNN architectures designed to address the issue of large and complex models being unsuitable for deployment on mobile or embedded devices. The fundamental building block of MobileNet is depthwise separable convolution, which significantly reduces the model size and improves inference speed while maintaining competitive performance. MobileNetV1 achieved an accuracy of 95.67% on the test set, MobileNetV2 achieved an accuracy of 93.86%, MobileNetV3_Small achieved an accuracy of 93.86%, and MobileNetV3_Large achieved an accuracy of 94.22%.

The EfficientNet model adopts a residual network architecture similar to ResNet, but incorporates several novel modules, such as the MBConv module and the SE module. Additionally, EfficientNet employs an adaptive network scaling method to automatically determine the network depth, width, and resolution, thereby improving model performance without increasing computational cost. EfficientNet consists of seven stages, each containing a set of MBConv modules and an SE module. Each MBConv module includes sub-modules such as depthwise convolution, expansion convolution, and projection convolution, which are used to extract features at different levels. The SE module reweights the feature channels to enhance the model’s representational power. EfficientNetB0 achieved a final accuracy of 95.31%, while EfficientNetB1 achieved a final accuracy of 92.78%.

**Figure 10:**
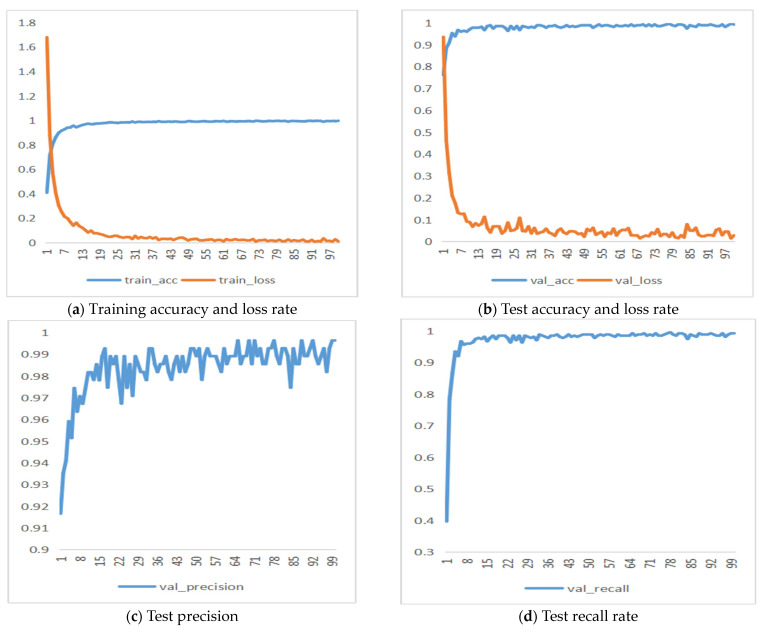
DenseNet201 model results.

**Figure 11:**
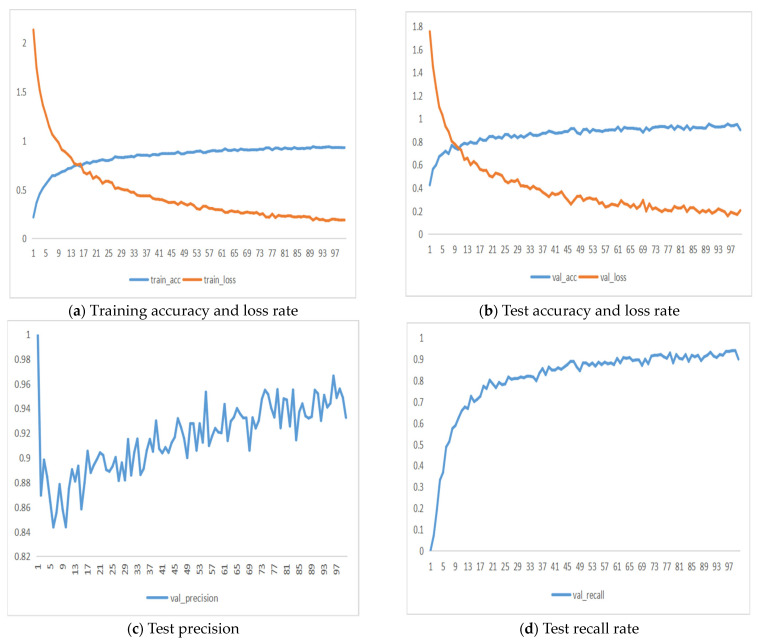
MobileNetV3_Small model results.

Compared to other classic convolutional neural network models, the DenseNet series models achieved the highest recognition accuracy. As shown in Figure 10 and Figure 11, the final accuracy of the DenseNet201 model was 99.28%, with a precision of 99.73%, recall of 99.68%, and F1 score of 99.70%, all of which were higher than those of other classic CNN models. The final accuracy of the MobileNetV3_Small model was 94.22%, with a precision of 93.99%, recall of 91.99%, and F1 score of 92.98%.

As shown in Table 5, the VGG model is relatively outdated and employs a deeper convolutional network, with an inference time of 40–50 ms per image. It has a large number of parameters and high computational complexity, resulting in slow inference on large datasets. The ResNet model has an inference time of 30–40 ms per image, with relatively slower inference, making it suitable for devices with moderate computational resources. The DenseNet model, with its densely connected structure, increases computational complexity, leading to an inference time of 45–60 ms per image and slower inference on large-scale datasets. The MobileNet series models are lightweight and fast in inference, making them suitable for resource-constrained environments such as mobile devices and embedded systems. The MobileNetV3_Small model has an inference time of 6 ms per image, offering lower latency and storage requirements compared to other convolutional neural networks. The EfficientNet model has an inference time of 10–22 ms per image. Though faster than DenseNet, it still requires more computational resources than the MobileNetV3_Small model.

As shown in Figure 10 and Figure 11, the DenseNet201 model achieved a final accuracy of 99.28%, precision of 99.73%, recall of 99.68%, and F1 score of 99.70%, outperforming other classic convolutional neural network models. In comparison, the MobileNetV3_Small model achieved a final accuracy of 94.22%, precision of 93.99%, recall of 91.99%, and F1 score of 92.98%. While the DenseNet series models achieved the highest recognition accuracy, the MobileNet series models, being more lightweight and offering faster model training, are better suited for classification tasks involving pork cuts and their freshness when considering both accuracy and computational efficiency.

### 3.2. The Role of Validation Data Enhancement

**Figure 12:**
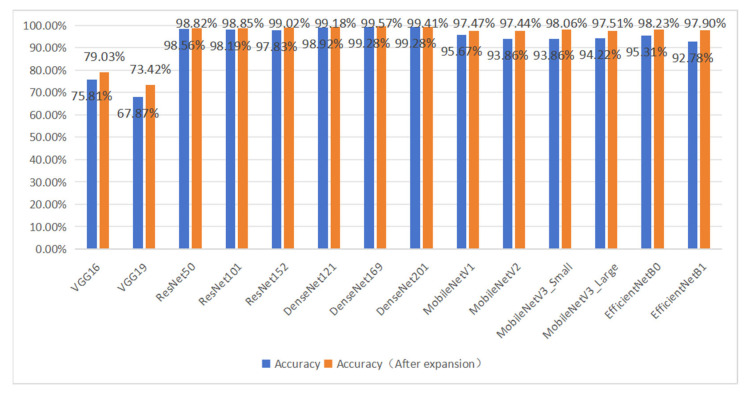
Comparison results of model accuracy before and after data enhancement.

**Figure 13:**
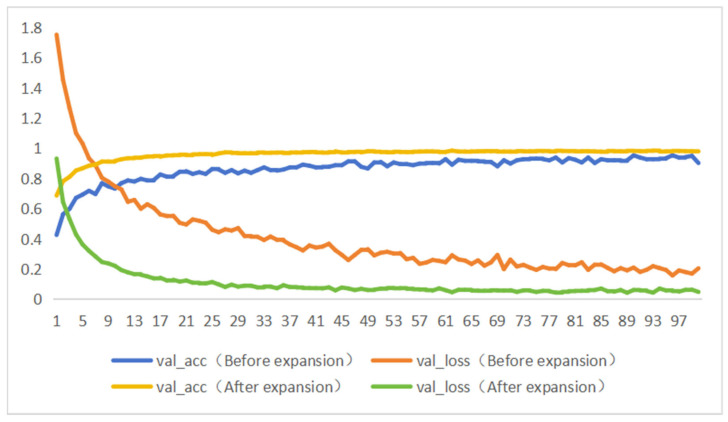
Change curves of accuracy and loss rate of test set before and after data expansion of MobileNetV3_Small model.

A comparative experiment was conducted to evaluate the impact of data augmentation on the recognition accuracy of CNN models. The results, shown in Table 6 and Figure 12, indicated that after augmenting the dataset, the accuracy, precision, recall, and F1 score of each model significantly improved. Figure 13 presents the change curves of accuracy and loss rate on the test set for the MobileNetV3_Small model before and after data augmentation. It is evident that when the CNN model is trained on the initial dataset, the training process fluctuates and is highly unstable. The final accuracy of the MobileNetV3_Small model on the test set was 93.86%. In comparison, the model trained on the augmented dataset achieved an accuracy of 98.06%, with a stabler training process and faster convergence. The initial dataset had a small number of image samples and lacked diversity in features, causing the network model to overly rely on a subset of features, leading to overfitting. Data augmentation simulated the real-world environment of various pork cuts, greatly increasing the number of images and enriching the diversity of features. Training the CNN model on the augmented dataset allowed the model to learn more features, improving its robustness and interference resistance in complex environments.

### 3.3. Results of Model Significance Analysis

The significance analysis of test set accuracy between various neural network models and MobileNetV3_Small is shown in Table 7. The VGG series (VGG16, VGG19) and DenseNet series (DenseNet121, DenseNet169, DenseNet201) models exhibited significant differences from MobileNetV3_Small, with *p*-values extremely small and well below 0.05, indicating statistically significant differences in performance for pork feature recognition. The ResNet series (ResNet50, ResNet152) also showed significant differences from MobileNetV3_Small. Although the *p*-value for ResNet50 was slightly larger (0.0037), it is still below 0.05, suggesting a significant difference. The MobileNet series (MobileNetV1, MobileNetV2, MobileNetV3_Large) also demonstrated significant differences from MobileNetV3_Small. Despite being part of the MobileNet family, the differences in their versions lead to variations in performance in certain aspects.

The *p*-value for ResNet101 was 0.0963, indicating no significant difference compared to MobileNetV3_Small. Although ResNet101 is a deeper model within the ResNet series, its performance on this task was not significantly different from that of MobileNetV3_Small. The EfficientNet series (EfficientNetB0 and EfficientNetB1) showed no significant difference compared to MobileNetV3_Small either. Despite EfficientNet being an efficient network architecture, there was no notable performance difference between the two on the tasks analyzed in this study.

### 3.4. Validating the Role of Added Attention Mechanisms

**Figure 14:**
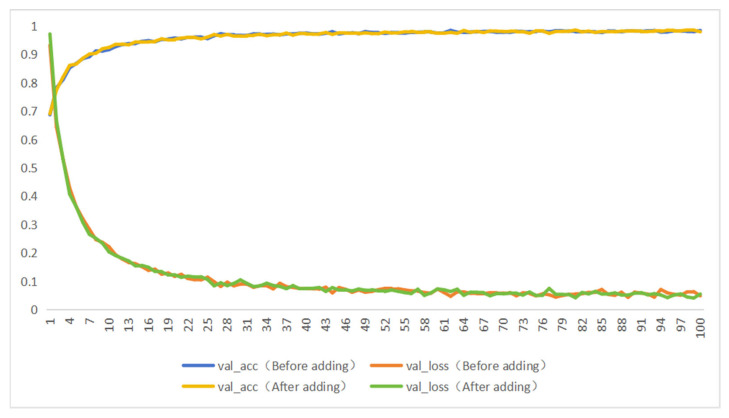
Change curves before and after adding the attention mechanism module.

As shown in Table 8 and Figure 14, the accuracy of the MobileNetV3_Small model on the test set increased from 98.06% to 98.59% after incorporating the attention mechanism, indicating an improvement in image classification accuracy. The attention mechanism allows the model to focus more on important features, thereby reducing the impact of noise. The precision improved from 99.56% to 99.71%, and the F1 score increased from 99.55% to 99.69%, demonstrating enhanced performance in handling imbalanced data. The attention mechanism can dynamically select and emphasize crucial features, leading to faster convergence during training. As a result, the required number of training epochs or iterations is reduced, improving training efficiency.

### 3.5. Comparison of Categories

The confusion matrix is a standard format for accuracy evaluation, and was used to visualize the recognition performance of the convolutional neural network (CNN) model for each class in this study. Each column of the confusion matrix represents a predicted class, and the total sum of each column indicates the total number of predictions for that class. Each row represents the true class, and the total sum of each row indicates the total number of instances of that class. The values within each column represent the number of true instances predicted to be in that particular class. Therefore, the confusion matrix provides an intuitive display of the model’s confusion across different classes.

**Figure 15:**
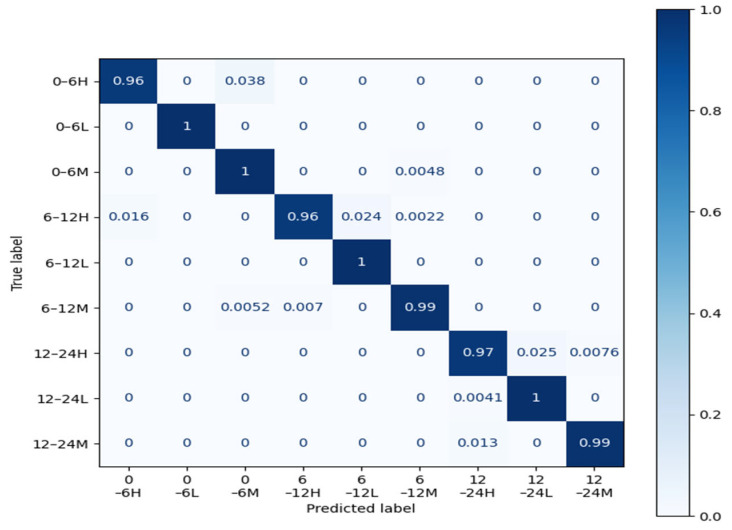
Confusion matrix diagram for the MobileNetV3_Small (add attention mechanism) model.

During classification, the shape and color similarities of pork cuts can lead to misclassification by the model. Additionally, the variation in freshness of the same cut can result in significant morphological differences, further decreasing the classification accuracy. Table 9 and Figure 15 present the final recognition results of the MobileNetV3_Small model on the test dataset. There is a certain degree of morphological similarity between pork belly and ham, especially in the distribution of fat and muscle tissue, which can easily lead to confusion due to their similar fat layers. Pork belly has a higher fat content, whereas ham typically contains more lean meat. Despite differences in the distribution of meat and fat, the visual model may struggle to fully distinguish between the two based on color and texture due to their similar appearance. Pork loin, on the other hand, has a relatively unique shape, being long and smooth, which makes it easier to distinguish from both pork belly and ham. The color of pork is influenced by several biological factors, including the hemoglobin content in muscle tissue, the degree of fat oxidation, and storage time. The color of fresh meat, characterized by red muscle and white fat, differs significantly from that of spoiled meat. After slaughter, the pH of pork changes as glycogen in the muscles is converted into lactic acid, lowering the pH. Fresh meat typically has bright colors and firm texture, whereas as freshness decreases, the meat becomes looser, with water loss or darkening of color. Over time, fat oxidation causes the fat layer of pork to change from white to yellow or brown, which directly affects the model’s classification of pork belly and ham. The experiments above show that the MobileNetV3_Small model achieves high recognition accuracy across nine different pork cut categories, including ham, pork loin, and pork belly.

### 3.6. Feature Visualization

Convolutional neural networks (CNNs) operate in an end-to-end manner, which results in relatively weak interpretability. To reveal and understand the mechanisms behind the learning and functioning of deep CNNs and to explore how the model learns features to differentiate between different classes, we employed a visualization technique based on gradient-weighted class activation mapping (Grad-CAM) to uncover the feature learning process of the model.

For deep convolutional neural networks, after multiple convolutions and pooling operations, the final convolutional layer contains the most informative spatial and semantic features. Grad-CAM utilizes the gradient information flowing into the last convolutional layer of the CNN to assign importance values to each neuron, aiding in specific attention decisions. The class activation heatmaps generated by Grad-CAM provide valuable insights into which regions of the input image contribute to the final classification decision made by the convolutional neural network.

**Figure 16:**
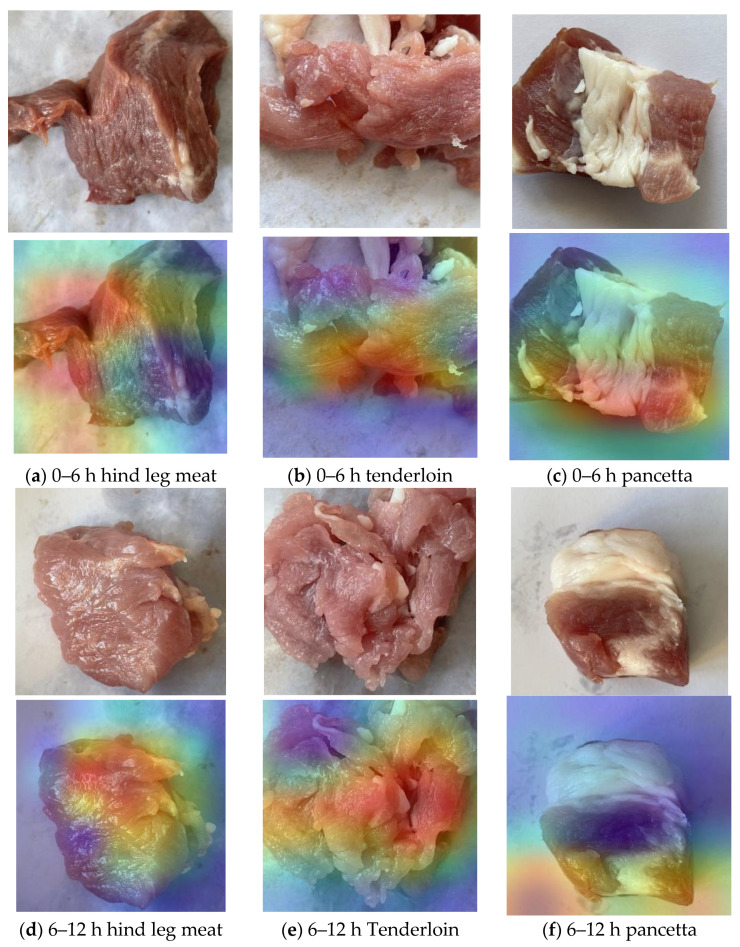

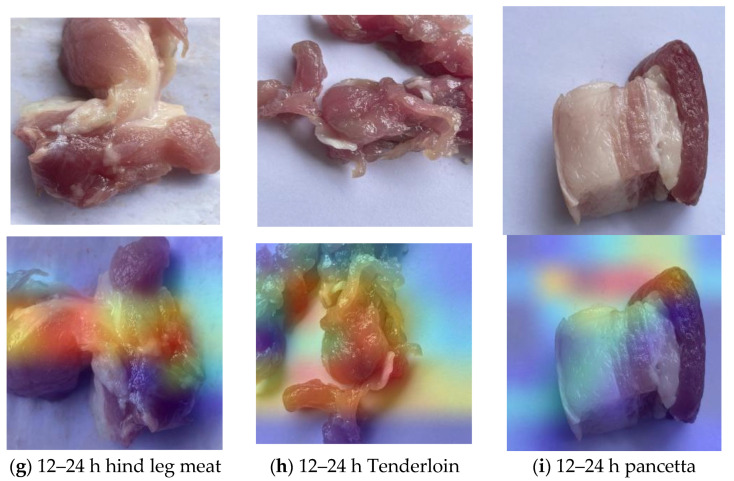
Raw images of the 9 classes and their corresponding class activation heat maps.

Different cuts of pork exhibit distinct biological structures and tissue compositions. The texture of the meat is typically determined by factors such as the arrangement of muscle fibers, fat distribution, and connective tissue. Figure 16 presents the original images and their corresponding class activation heatmaps for nine categories, including ham, pork loin, and pork belly.

Based on the visualization results, the hind leg meat is primarily composed of long, strip-shaped muscle fibers, with relatively less fat, and the fat distribution is uneven, resulting in a rough texture. The heatmap reveals different activation patterns between the regions with less fat and the muscle areas. In contrast, the belly pork contains more fat tissue, causing the muscle fibers to be arranged more loosely with a distinct texture. Belly pork is especially characterized by the significant layer distribution between fat and meat, exhibiting strong layering and complex structure. In the heatmap, the fat layers show different thermal zones, with areas rich in fat displaying lower activation, indicating a softer structure. The tenderloin has a loose fiber arrangement with minimal connective tissue and a more delicate texture. It contains relatively low but evenly distributed fat, and the heatmap reflects the fine texture of the fibers, with slight fat deposition possible. Fresh meat retains its protein structure, maintaining tightly arranged fibers, and the activation heatmap shows a more uniform muscle fiber distribution, with higher heat in the muscle fiber regions and less fat. Extended storage leads to fat oxidation, and the heatmap reveals less activity in the fat regions, indicating a reduction in fat quality. The texture regions of the pork cuts are activated, suggesting that the model has extracted the features of the pork texture regions and used them as the basis for the final classification decision. These experimental results demonstrate that the MobileNetV3_Small model effectively extracts features from different pork cuts, achieving accurate classification of pork cuts at various stages of storage.

### 3.7. Model Limitation Analysis

Lighting is a crucial factor influencing the visual appearance of food. Variations in ambient light, such as low light or strong shadows, can cause pork to appear differently, even if its freshness has not changed. Convolutional neural networks (CNNs) are sensitive to pixel intensity variations induced by lighting shifts. Inconsistent lighting can prevent a model from accurately recognizing subtle features related to pork freshness, such as color and texture. Although techniques like data augmentation can help CNNs generalize under varying lighting conditions, achieving robustness to all types of lighting variations remains a challenge, particularly when dealing with extreme lighting scenarios (e.g., strong backlighting or low-light environments).

The quality of the camera also plays a significant role in the accuracy of CNN models. Low-resolution cameras often fail to capture enough detail to distinguish between fresh and spoiled pork. This is especially critical when detecting subtle signs of spoilage, such as slight discoloration or texture changes. The freshness of pork can also be influenced by environmental factors such as temperature and humidity, which may not be directly visible in the images, but can subtly affect the appearance and texture of the meat. Overcoming these challenges requires ongoing research and innovation in applying deep learning to food quality inspection.

### 3.8. Model Deployment

**Figure 17:**
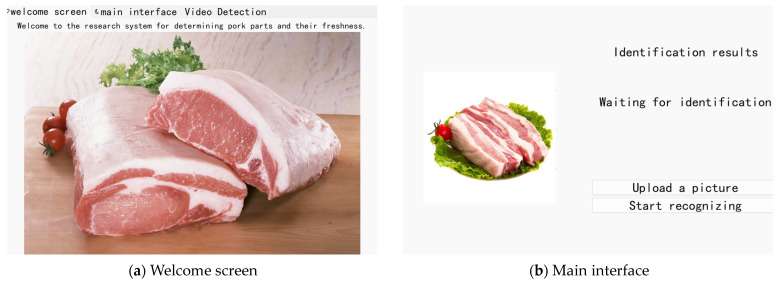

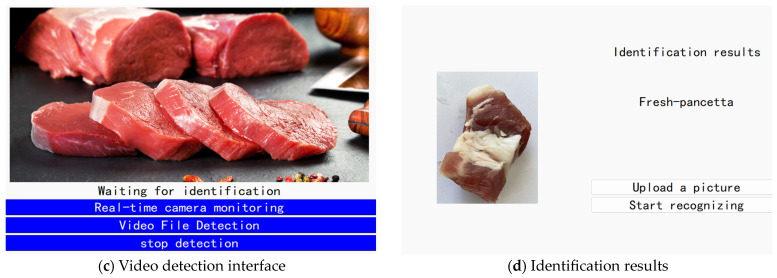
GUI interface for the study of pork parts and their freshness.

This study designed a GUI interface for pork cut and freshness classification based on the MobileNetV3_Small model, as shown in Figure 17. The interface primarily includes the following components.

1. Layout and structure of the main interface: The GUI interface includes a menu bar with various functional options, such as the main interface, video detection, and more. It also features a primary workspace that displays real-time visual feedback and processing results.
2. Controls and visual feedback: The controls and visual feedback on the interface are primarily used for interaction and displaying processing results. The image display area shows the detected pork images or video stream. The cut freshness recognition results display the identification outcomes for different pork cuts and their freshness levels.
3. User interaction and operation: This section describes how users interact with and operate the interface. Image or video input details how pork images or videos are imported for processing. The functional buttons include options such as “Upload Image”, “Start Recognition”, “Camera Detection”, “Video File Detection”, and “Stop Detection”.
4. The interface design ensures alignment with user habits and intuitiveness, with clear labels and instructions, including the naming and functional descriptions of interface elements. This ensures that users can accurately understand the meaning and impact of each operation. The feedback mechanism provides clear operational feedback, including success prompts, error messages, etc., helping users quickly comprehend the results of their actions.

Based on the above description, the GUI design for the pork cut and freshness determination method using computer vision can be clearly understood, thereby facilitating a better evaluation of its functionality and potential applications.

The GUI interface displays the results of the computer vision algorithm, such as pork images, labeling different cuts, and assessing freshness. This real-time feedback helps users intuitively understand the execution and analysis results of the algorithm. It enhances the user experience by making the operation more intuitive and convenient. Users can easily upload pork images or videos through the interface and view detailed processed information and evaluation results. The interface integrates functional buttons and parameter adjustment options, allowing users to control the initiation, termination, and parameter tuning of the algorithm for personalized analysis and result presentation. It may also include data management features, such as recording data from each analysis, saving results, and maintaining a history of previous analyses. These features are highly beneficial for experimental reproducibility, result comparison, and tracking the analysis process.

The design of the GUI interface enables the broader application of computer vision-based methods for pork cut classification and freshness assessment in areas such as meat production, supply chain management, and meat safety monitoring. Through an intuitive interface, these technologies become more accessible and easier to implement. This not only enhances the practicality and usability of computer vision techniques for pork freshness determination but also promotes the development and application of related research. It plays a crucial role in advancing meat safety and quality monitoring.

## 4. Conclusions

This study compared five convolutional neural network models—VGGNet, ResNet, DenseNet, MobileNet, and EfficientNet—based on different cuts and freshness levels of pork. The MobileNetV3_Small model, which is more lightweight, was selected for the task. After incorporating an attention mechanism, the final accuracy reached 99.71%, with an overall precision of 98.59%. These results indicate that the MobileNetV3_Small model demonstrates excellent learning capability in recognizing and classifying different cuts and freshness levels of pork. Additionally, the model was integrated with the PYQT5 framework to develop a real-time GUI for pork identification and quality assessment. The findings of this study provide valuable insights for the development of pork detection and identification systems and offer technical support for ongoing research and innovation in the application of computer vision for food quality inspection.

## Figures and Tables

**Table 1:** Initial dataset information.

Pork Part	Number of Training Sets/Test Sets/Validation Sets	Total Number
0–6 h hind leg meat	237/29/30	296
0–6 h tenderloin	138/17/17	172
0–6 h pancetta	158/19/20	197
6–12 h hind leg meat	332/41/42	415
6–12 h tenderloin	167/20/21	208
6–12 h pancetta	421/52/53	526
12–24 h hind leg meat	292/36/36	364
12–24 h tenderloin	183/22/23	228
12–24 h pancetta	281/35/35	351
total number	2209/271/277	2757

**Table 2:** Expanded dataset.

Pork Part	Number of Training Sets/Test Sets/Validation Sets	Total Number
0–6 h hind leg meat	2607/319/330	3256
0–6 h tenderloin	1518/187/187	1892
0–6 h pancetta	1738/209/220	2167
6–12 h hind leg meat	3652/451/462	4565
6–12 h tenderloin	1837/220/231	2288
6–12 h pancetta	4631/572/583	5786
12–24 h hind leg meat	3212/396/396	4004
12–24 h tenderloin	2013/242/253	2508
12–24 h pancetta	3091/385/385	3861
total number	24299/2981/3047	30,327

**Table 3:** Hardware and software environment configuration.

Configuration Items	Item Value
Operating System	Microsoft Windows 11 Home Edition
CPU	AMD Ryzen 7 5800H with Radeon Graphics
GPU	GeForce RTX 3070
Available Video Memory	8192MiB
Programming Languages	Python
Programming IDE	Pycharm
Deep Learning Framework	TensorFlow, Keras
Model Deployment Framework	Django, PYQT5

**Table 4:** CNN model results.

Model	Accuracy	Precision	Recall	F1 Score
VGG16	75.81%	84.66%	57.22%	68.29%
VGG19	67.87%	80.53%	45.68%	58.29%
ResNet50	98.56%	97.91%	97.78%	97.84%
ResNet101	98.19%	98.46%	98.28%	98.37%
ResNet152	97.83%	99.00%	98.91%	98.95%
DenseNet121	98.92%	99.46%	99.46%	99.46%
DenseNet169	99.28%	99.50%	99.46%	99.48%
DenseNet201	99.28%	99.73%	99.68%	99.70%
MobileNetV1	95.67%	96.40%	95.70%	96.05%
MobileNetV2	93.86%	96.98%	96.06%	96.52%
MobileNetV3_Small	93.86%	93.99%	91.99%	92.98%
MobileNetV3_Large	94.22%	97.02%	95.88%	96.45%
EfficientNetB0	95.31%	97.26%	96.29%	96.77%
EfficientNetB1	92.78%	96.00%	94.48%	95.23%

**Table 5:** CNN model size and computational speed results.

Model	Model Size	Reasoning Time per Image
VGG16	56.16 MB	43 ms
VGG19	76.42 MB	50 ms
ResNet50	90.38 MB	31 ms
ResNet101	163.50 MB	36 ms
ResNet152	223.83 MB	40 ms
DenseNet121	27.74 MB	45 ms
DenseNet169	49.47 MB	52 ms
DenseNet201	71.37 MB	58 ms
MobileNetV1	16.43 MB	10 ms
MobileNetV2	8.97 MB	11 ms
MobileNetV3_Small	4.13 MB	6 ms
MobileNetV3_Large	12.10 MB	10 ms
EfficientNetB0	15.93 MB	18 ms
EfficientNetB1	25.77 MB	22 ms

**Table 6:** Model results after data enhancement.

Model	Accuracy	Precision	Recall	F1 Score
VGG16	79.03%	89.78%	81.88%	85.65%
VGG19	73.42%	84.93%	69.09%	76.20%
ResNet50	98.82%	99.88%	99.88%	99.88%
ResNet101	98.85%	99.89%	99.89%	99.89%
ResNet152	99.02%	99.88%	99.88%	99.88%
DenseNet121	99.18%	99.97%	99.97%	99.97%
DenseNet169	99.57%	99.87%	99.86%	99.86%
DenseNet201	99.41%	99.91%	99.90%	99.90%
MobileNetV1	97.47%	99.79%	99.79%	99.79%
MobileNetV2	97.44%	99.79%	99.78%	99.78%
MobileNetV3_Small	98.06%	99.56%	99.54%	99.55%
MobileNetV3_Large	97.51%	99.82%	99.81%	99.81%
EfficientNetB0	98.23%	99.79%	99.79%	99.79%
EfficientNetB1	97.90%	99.79%	99.79%	99.79%

**Table 7:** Significant difference analysis of different models (based on *p*-values).

Model	*p*-Value	Comparison of Results
VGG16	5.10 × 10^−11^	*p*-value < 0.05
VGG19	5.57 × 10^−14^	*p*-value < 0.05
ResNet50	0.0037	*p*-value < 0.05
ResNet101	0.0963	*p*-value > 0.05
ResNet152	8.87 × 10^−5^	*p*-value < 0.05
DenseNet121	1.50 × 10^−6^	*p*-value < 0.05
DenseNet169	5.69 × 10^−6^	*p*-value < 0.05
DenseNet201	1.17 × 10^−5^	*p*-value < 0.05
MobileNetV1	0.0168	*p*-value < 0.05
MobileNetV2	0.0119	*p*-value < 0.05
MobileNetV3_Large	0.0005	*p*-value < 0.05
EfficientNetB0	0.3383	*p*-value > 0.05
EfficientNetB1	0.0972	*p*-value > 0.05

**Table 8:** Results before and after adding the attention mechanism module.

Model	Accuracy	Precision	Recall	F1 Score
MobileNetV3_Small	98.06%	99.56%	99.54%	99.55%
MobileNetV3_Small (add attention mechanism)	98.59%	99.71%	99.68%	99.69%

**Table 9:** Confusion matrix for the MobileNetV3_Small (add attention mechanism) model.

	Form	Forecast Category									Precision	Recall	F1 Score
		0–6 H	0–6 L	0–6 M	6–12 H	6–12 L	6–12 M	12–24 H	12–24 L	12–24 M			
**True Category**	**0–6 H**	307	0	12	0	0	0	0	0	0	97.77%	96.24%	97.00%
	**0–6 L**	0	187	0	0	0	0	0	0	0	100%	100%	100%
	**0–6 M**	0	0	208	0	0	1	0	0	0	93.27%	99.52%	96.29%
	**6–12 H**	7	0	0	432	11	1	0	0	0	99.08%	95.79%	97.41%
	**6–12 L**	0	0	0	0	220	0	0	0	0	95.24%	100%	97.56%
	**6–12 M**	0	0	3	4	0	565	0	0	0	99.65%	98.78%	99.21%
	**12–24 H**	0	0	0	0	0	0	383	10	3	98.46%	96.72%	97.58%
	**12–24** **L**	0	0	0	0	0	0	1	241	0	96.02%	99.59%	97.77%
	**12–24 M**	0	0	0	0	0	0	5	0	380	99.22%	98.70%	98.96%

## Data Availability

The authors do not have permission to share the data.
